# Correction: Plasma Membrane Is the Site of Productive HIV-1 Particle Assembly

**DOI:** 10.1371/journal.pbio.3000078

**Published:** 2018-11-16

**Authors:** The PLOS Biology Staff

## Notice of republication

This article was republished on November 2, 2018, to replace the supporting information file S3 Video, which had previously been unavailable for download. The caption remains the same. The publisher apologizes for this error.
